# Plasma UCHL1, GFAP, Tau, and NfL Are Not Different in Young Healthy Persons With Mild COVID-19 Symptoms Early in the Pandemic: A Pilot Study

**DOI:** 10.1089/neur.2023.0014

**Published:** 2023-05-15

**Authors:** Matthew J. Rogatzki, Rachel E. Szeghy, Nina L. Stute, Valesha M. Province, Marc A. Augenreich, Jonathon L. Stickford, Abigail S.L. Stickford, Erik D. Hanson, Stephen M. Ratchford

**Affiliations:** ^1^ Department of Public Health and Exercise Science, Appalachian State University, Boone, North Carolina, USA.; ^2^ School of Kinesiology, Auburn University, Auburn, Alabama, USA.; ^3^ Department of Cardiovascular and Metabolic Sciences, The Cleveland Clinic Foundation, Cleveland, Ohio, USA.; ^4^ Department of Nutrition and Exercise Physiology, University of Missouri, Columbia, Missouri, USA.; ^5^ Department of Exercise and Sport Science, University of North Carolina, Chapel Hill, North Carolina, USA.

**Keywords:** biomarkers, COVID-19, GFAP, mild symptoms, NfL, plasma, Tau, UCHL1

## Abstract

Elevated levels of brain injury biomarkers have been found primarily in middle-aged or older persons experiencing moderate-to-severe COVID-19 symptoms. However, there is little research in young adults, and there is concern that COVID-19 causes brain injury even in the absence of moderate-to-severe symptoms. Therefore, the purpose of our study was to investigate whether neurofilament light (NfL), glial fibrillary acidic protein (GFAP), tau, or ubiquitin carboxyl-terminal esterase L1 (UCHL1) are elevated in the plasma of young adults with mild COVID-19 symptoms. Twelve participants diagnosed with COVID-19 had plasma collected 1, 2, 3, and 4 months after diagnosis to determine whether NfL, GFAP, tau, and UCHL1 concentrations increased over time or whether plasma concentrations were elevated compared with COVID-19-naïve participants. We also compared plasma NfL, GFAP, tau, and UCHL1 concentrations between sexes. Our results showed no difference between NfL, GFAP, tau, and UCHL1 concentrations in COVID-19-naïve participants and COVID-19-positive participants at any of the four time points (*p* = 0.771). Within the COVID-19-positive participants, UCHL1 levels were higher at month 3 after diagnosis compared to month 1 or month 2 (*p* = 0.027). Between sexes, females were found to have higher UCHL1 (*p* = 0.003) and NfL (*p* = 0.037) plasma concentrations compared to males, whereas males had higher plasma tau concentrations than females (*p* = 0.024). Based on our data, it appears that mild COVID-19 in young adults does not increase plasma NfL, GFAP, tau, or UCHL1.

## Introduction

Severe acute respiratory syndrome coronavirus 2 (SARS-CoV-2) causes corona virus disease 2019 (COVID-19), which is an upper respiratory disease first discovered in December 2019 in Wuhan, China.^1,2^ In addition to respiratory problems, research has shown that COVID-19 can result in neurodegeneration^2–10^ and neurological disorders.^11–18^ Investigating biomarkers of brain injury is one way the effect of COVID-19 on the central nervous system has been examined. Common biomarkers of brain injury include neurofilament light (NfL),^19^ glial fibrillary acidic protein (GFAP),^20^ tau,^21^ and ubiquitin carboxyl-terminal esterase L1 (UCHL1).^22^

Research investigating blood biomarkers of brain injury in SARS-CoV-2-infected persons have largely been conducted in patients admitted to the hospital and compared to controls not infected with SARS-CoV-2 during the pandemic^23–25^ or comparing the severity of COVID-19 symptoms among SARS-CoV-2-infected persons.^26–28^ Only three studies have specifically investigated participants with mild symptoms.^23,26,29^ Given that there is a paucity of research investigating young adults infected with SARS-CoV-2 and experiencing mild symptoms, we conducted our research out of concern that COVID-19 may be causing brain injury in SARS-CoV-2-infected, otherwise young and healthy persons, even in the absence of moderate-to-severe symptoms. Because this was an exploratory study in persons not hospitalized with minor symptoms from COVID-19, we limited our investigation to the plasma biomarkers tau, GFAP, NfL, and UCHL1 and compared their biomarker levels to a small cohort of healthy controls whose plasma was obtained before the beginning of the COVID-19 outbreak in North Carolina. We included NfL and GFAP because these were the two most common biomarkers found to be elevated in COVID-19 participants.^23–32^ Tau was included in our study because only two other studies investigated this protein in the blood^31,32^ and tau has been shown to indicate brain injury.^33^ UCHL1 was included because one study found increased UCHL1 plasma levels in COVID-19 patients compared to pre-COVID-19 controls^31^ and because UCHL1 has been shown to indicate brain injury.^34^ We hypothesized that the SARS-CoV-2-positive participants would have higher plasma levels of GFAP, UCHL1, NfL, and tau when compared with SARS-CoV-2-naïve participants and that these elevated levels would decrease over time.

## Methods

All procedures were approved by the Appalachian State University Institutional Review Board (IRB_20–0304 and IRB_20-0151). Participants provided written informed consent before testing. Residents of the greater Boone, North Carolina area were solicited through mass communication and flyers to participate in the study if they tested positive for SARS-CoV-2 by the standard PCR test between October 2020 and January 2021. Potential participants who tested positive for SARS-CoV-2 > 4 weeks before screening were deemed ineligible to participate. Healthy control participants were retrospectively studied from Elon, North Carolina in February 2020 before any known cases of SARS-CoV-2 were present in the state of North Carolina.^35^ These participants were recruited through mass communication for another study.^36^

**FIG. 1.**
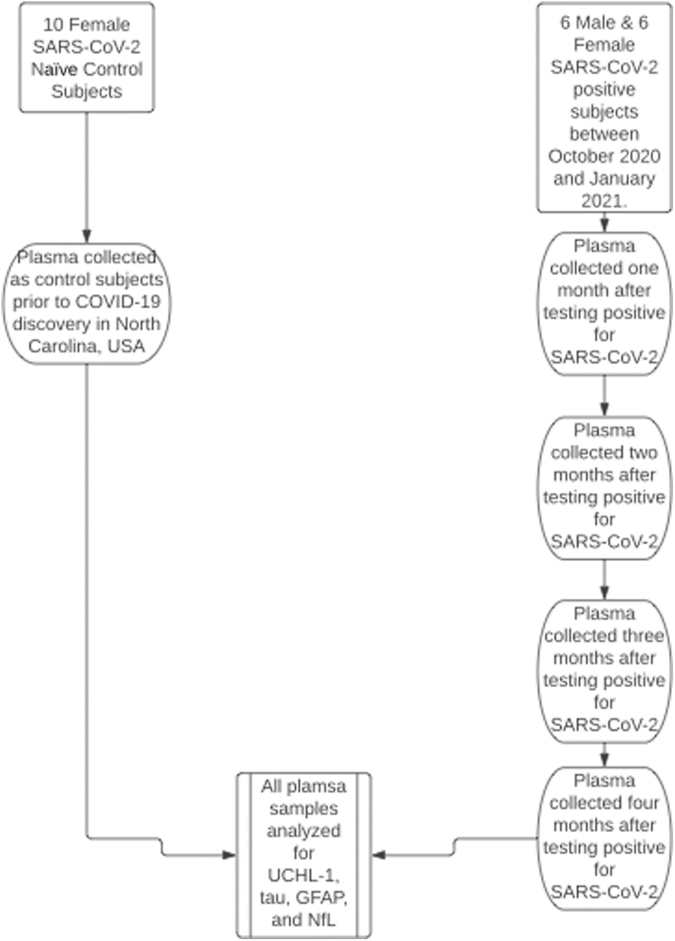
Schematic displaying the brief methodology of data collection. GFAP, glial fibrillary acidic protein; NfL, neurofilament light; UCHL1, ubiquitin carboxyl-terminal esterase L1.

Participants in both the SARS-CoV-2-positive and SARS-CoV-2-naïve groups were excluded from the study if they had a history of neurological disease or were participating in a contact sport or other activities that could result in traumatic brain injury (TBI). All participants were recreationally active healthy young adults free from cardiovascular, metabolic, or chronic neurological diseases. A more specific health history, along with race/ethnicity data, for participants in the study can be seen in Supplementary Table S1. All SARS-CoV-2-positive participants were at a score of 1 (ambulatory mild disease, asymptomatic, or viral DNA detected) or 2 (ambulatory mild disease, symptomatic, or independent) on the World Health Organization clinical progression scale.^37^ None of the participants had received a COVID-19 vaccination at the time of data collection, nor did they receive treatment for COVID-19 over the course of the study. However, during the data collection period of the SARS-CoV-2 participants, 3 male participants received vaccinations. In order to allow any possible vaccination symptoms or effects to subside, 2 weeks were required between vaccination and the following blood draw. Neither biomarker concentrations nor symptoms appeared to be affected by these vaccinations. During the time of the study, SARS-CoV-2-positive participants were quarantining to the best of their ability and masking. Further, the time frame of the study occurred when non-essential businesses were closed. These practices minimized the risk of secondary exposure to SARS-CoV-2 during the time of the study. A schematic description of our study design can be seen in Figure 1.

Participants who tested positive for SARS-CoV-2 were given a brief survey associated with mental clarity during each visit. The instructions given to each participant for the survey were as follows: “Write down your score 0 to 100 how severe your COVID-19 symptoms are today. Use only whole numbers to indicate your score for each symptom.” For survey results during each visit, see Table 1.

**Table 1.** SARS-CoV-2 Survey Results for Each SARS-CoV-2-Positive Participant at Each Month

	Dizziness/vertigo				Headache				Anosmia			
Participant	V1	V2	V3	V4	V1	V2	V3	V4	V1	V2	V3	V4
01-Male	0	0	0	0	0	0	0	0	0	0	0	0
02-Male	0	0	0	0	0	0	0	0	55	30	30	0
03-Male	0	0	0	0	0	0	0	0	0	0	0	0
04-Male	0	0	0	0	0	0	0	0	0	0	0	0
05-Male	0	0	0	0	0	0	0	0	0	0	0	0
06-Male	0	0	0	0	0	0	0	0	0	0	0	0
07-Female	0	0	0	0	0	0	0	0	10	3	3	2
08-Female	10	0	0	0	0	0	0	0	0	0	0	0
09-Female	20	0	0	N/A	0	0	20	N/A	0	0	0	N/A
10-Female	0	0	0	0	0	10	30	20	10	10	20	30
11-Female	0	0	0	0	0	0	15	10	50	60	30	30
12-Female	0	0	0	0	40	20	10	0	70	0	10	0

Participants reported symptoms during each visit on a scale of 0–100 based on severity of symptoms, with 0 being absence of symptoms and 100 being the most extreme severity. V1 = visit one (∼1 month post-COVID-19 diagnosis), V2 = visit 2 (∼2 months post-COVID-19 diagnosis), V3 = visit 3 (∼3 months post-COVID-19 diagnosis), and V4 = visit 4 (∼4 months post-COVID-19 diagnosis). 04-Male received his first dose of the Moderna vaccine between visit 1 and visit 2 and received his second dose of the Moderna vaccine between visit 3 and visit 4. 05-Male received his first dose of the Moderna vaccine between visit 1 and visit 2 and received his second dose of the Moderna vaccine between visit 2 and visit 3. 06-Male received his first dose of the Pfizer vaccine between visit 1 and visit 2 and received his second dose of the Pfizer vaccine between visit 3 and visit 4. None of the other participants received a vaccine during the study.

N/A, not available.

SARS-CoV-2-positive participants came into the laboratory for four total study visits at approximately 1-month intervals for 4 months. All participants arrived for testing at approximately the same time of day each visit, having fasted for at least 4 h, having abstained from caffeine for 12 h, and having abstained from exercise and alcohol for 24 h. Venous blood was collected from a prominent antecubital vein into a 4-mL vacutainer containing sodium-heparin in a thermoneutral environment after 20 min of supine rest. All blood was centrifuged immediately after collection at 3500 RPM (2630*g*) for 10 min at 4°C (ThermoScientific Sorvall Legend RT+ refrigerated centrifuge; ThermoFisherScientific, Inc., Walthum, MA).

### Biomarker analysis

All plasma protein concentration measurements were performed in a laboratory at the University of North Carolina–Chapel Hill by a collaborative investigator who was blinded to the data. The Simoa^®^ Human Neurology 4-Plex A assay (Quanterix Corporation, Billerica, MA) was used to analyze serum samples and was run on an HD-1 analyzer (Quanterix Corporation), following manufacturer instructions. This assay can detect UCHL1 in plasma samples from a range of 0–40,000 pg⋅mL^−1^, GFAP in plasma samples from a range of 0–4000 pg⋅mL^−1^, tau in plasma samples from a range of 0–400 pg⋅mL^−1^, and NfL in plasma samples from a range of 0–2000 pg⋅mL^−1^. A single batch of reagents were used. None of the plasma samples were below the level of detection for the assay, and all samples were analyzed in duplicate. The average intra-assay coefficient of variation was 3.7% for UHCL1, 4.0% for GFAP, 7.3% for tau, and 8.2% for NfL.

### Statistical analyses

A multiple imputation approach using five imputations and taking the mean of the five imputations was used to substitute for all missing data points (1 participant missed the fourth visit; therefore, multiple imputation was only performed on one missing data point). The original data set was compared to the data set after multiple imputation using a paired *t*-test. Outliers were identified using the *Z*-score method, with any data above 3 or below −3 further investigated to determine whether the data should be removed from the data set. Normality was tested for sex, age, weight, and height using a Shapiro-Wilk normality test. Homogeneity of variance was tested for sex, age, weight, and height using a Levene statistic. Although the assumption for normality was not met for sex, age, weight, and height within the SARS-CoV-2-positive and SARS-CoV-2-naïve group, a multi-variate analysis of variance (MANOVA) was used to compare means between groups because the MANOVA has been found to outperform the non-parametric test when only the assumption of normal distribution is violated.^38^ Normality was tested for each protein at each visit and each group using a Shapiro-Wilk normality test. Homogeneity of variance was tested for each protein at each visit and each group using a Levene statistic. When multi-variate normality was met for all visits, a repeated-measures analysis of variance (RMANOVA) was used to compare means among visits, and partial eta squared (η^2^) was used to determine effect size. If significance was found using RMANOVA, a Bonferroni correction factor was used for pair-wise comparisons. When multi-variate normality was not met, a Friedman test was used to compare medians among visits and Kendall's *W* was used to determine effect size. When significance was found, a Bonferroni correction factor was used for pair-wise comparisons. A MANOVA was used to compare protein concentrations in SARS-CoV-2-naïve participants to each visit of SARS-CoV-2-positive participants. Alpha was set at 0.05 to determine statistical significance. All statistical analyses were generated using Statistical Package for the Social Sciences (SPSS) software (version 28; SPSS, Inc., Chicago, IL).

## Results

All 12 SARS-CoV-2-positive participants returned to the laboratory for four visits, with the exception of 1 participant who missed their fourth visit. Using the paired *t*-test to compare means between the original data set and the data set after multiple imputation, there was a standard deviation (SD) of 0, and therefore the test could not be performed. This shows that there was no difference between these two data sets; therefore, we used the data set derived from multiple imputation for all statistical analyses. There was one outlier in our data set for age and body mass. Coincidentally, the same participant that was the outlier for age (33 years old) was also the outlier for body mass (120.2 kg). Given that this participant did not have outlier data for any of the proteins, the participant was left in the data set. The MANOVA of anthropometric data showed a significant difference for the SARS-CoV-2-positive and SARS-CoV-2-naïve participants by group (*F*_(3,17)_ = 12.341, *p* < 0.001, η^2^ = 0.685) and sex (*F*_(3,17)_ = 7.971, *p* = 0.002, η^2^ = 0.584). Significance between groups was for age (*p* = 0.003), with the SARS-CoV-2-naïve participants having a mean average difference of being 4 years older than the SARS-CoV-2-positive participants. Sex differences were found only for height (*p* < 0.001), with males having a mean difference of being 21.25 cm taller than females. All anthropometric data are presented in Table 2.

**Table 2.** Anthropometric Data for All Participants in Each Group Along with Anthropometric Data Separated by Sex

Group	*N*	Age (years)	Height (cm)	Mass (kg)
SARS-CoV-2 positive	12	21.0 (1.0)	175.05 (11.33)	65.41 (15.25)
Males	6	22.0 (1.0)	184.15 (5.73)^+^	64.43 (22.26)
Females	6	21.0 (1.0)	165.95 (7.12)	66.38 (3.77)
SARS-CoV-2 naïve	10	25.0 (3.0)	161.07 (7.33)	65.16 (6.29)
Males	0	N/A	N/A	N/A
Females	10	25.0 (3.0)^*^	161.07 (7.33)	65.16 (3.77)

Data analyzed using a multi-variate analysis of variance and reported as mean (SD).

^*^ Significantly different compared to the SARS-CoV-2-positive group (*p* = 0.003); ^+^significantly different compared to females (*p* < 0.001).

cm, centimeters; kg, kilogram; N/A, not applicable; SD, standard deviation.

**FIG. 2.**
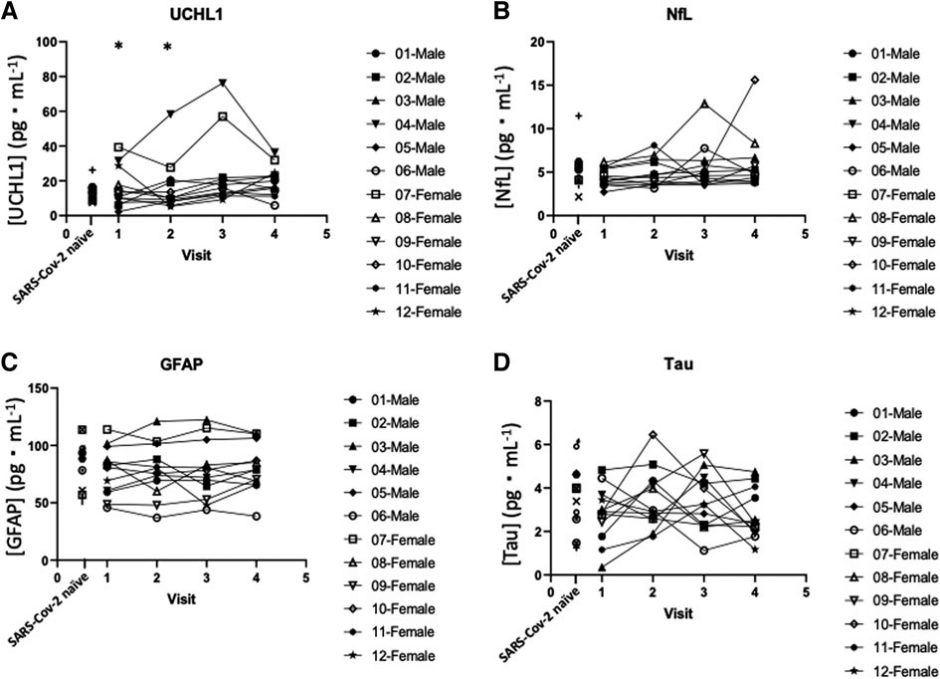
Line graphs of individual plasma biomarker concentration in COVID-19-positive participants over the four visits of data collection as well as SARS-CoV-2-naïve participants. (**A**) Ubiquitin carboxyl-terminal esterase L1 (UCHL1) plasma concentration over four visits. (**B**) Neurofilament light (NfL) plasma concentration over four visits. (**C**) Glial fibrillary acidic protein (GFAP) plasma concentration over four visits. (**D**) Tau plasma concentration over four visits. *Significantly different compared to visit 3 (*p* = 0.027).

Data for UCHL1 can be found in Table 3, data for NfL can be found in Table 4, data for GFAP can be found in Table 5, and data for tau can be found in Table 6. No differences were found among the four visits for GFAP (*F*_(3,33)_ = 0.239, *p* = 0.869, η^2^ = 0.021), tau (*F*_(3,33)_ = 0.989, *p* = 0.410, η^2^ = 0.082), or NfL (test statistic = 6.300, *p* = 0.098, Kendall's *W* = 0.184). However, significant differences were found among the four visits for plasma UCHL1 concentration (test statistic = 13.200, *p* = 0.004, Kendall's *W* = 0.367). To determine between which visits significance was found, a related-samples Friedman test was performed with a Bonferroni correction factor, which showed the significance (*p* = 0.027) between visits 1 and 3, with visit 3 having a median difference of being 4.85 pg⋅mL^−1^ higher than visit 1; and significance (*p* = 0.027) between visits 2 and 3, with visit 3 having a median difference of being 6.83 pg⋅mL^−1^ higher than visit 2. Individual biomarker data for each participant at each visit can be seen in Figure 2.

**Table 3.** UCHL1 Plasma Concentration at Each Visit

		UCHL1 (pg⋅mL^−1^)			
Group	*N*	Visit 1	Visit 2	Visit 3	Visit 4
SARS-CoV-2 positive	12	12.04 (Q1 = 9.38, Q3 = 20.39)^*^	10.06 (Q1 = 8.25, Q3 = 19.45)^*^	16.89 (Q1 = 12.74, Q3 = 21.21)	18.28 (Q1 = 14.56, Q3 = 23.15)
Males	6	16.04 (Q1 = 11.66, Q3 = 28.09)	15.10 (Q1 = 9.10, Q3 = 25.86)	18.75 (Q1 = 16.42, Q3 = 47.71)	21.17 (Q1 = 13.30, Q3 = 29.58)
Females	6	8.77 (Q1 = 6.43, Q3 = 12.51)	8.77 (Q1 = 8.15, Q3 = 12.46)	13.16 (Q1 = 12.27, Q3 = 19.07)	16.24 (Q1 = 15.49, Q3 = 21.16)
SARS-CoV-2 naïve	10	12.31 (Q1 = 10.03, Q3 = 15.72)	N/A	N/A	N/A
Males	0	N/A	N/A	N/A	N/A
Females	10	12.31 (Q1 = 10.03, Q3 = 15.72)	N/A	N/A	N/A

Data analyzed using a Friedman test for within the SARS-CoV-2-positive group analyses and using a multi-variate analysis of variance for between the SARS-CoV-2-positive and SARS-CoV-2-naïve group analyses. All data presented as median (Q1, Q3). *N* = number of participants, Q1 = 1st quartile, and Q3 = 3rd quartile,

^*^ Significantly different (*p* < 0.05) compared to visit 3.

UCHL1, ubiquitin carboxyl-terminal esterase L1; N/A, not available.

**Table 4.** NfL Plasma Concentration at Each Visit

		NfL (pg⋅mL^−1^)			
Group	*N*	Visit 1	Visit 2	Visit 3	Visit 4
SARS-CoV-2 positive	12	4.18 (Q1 = 3.71, Q3 = 5.41)	4.48 (Q1 = 3.57, Q3 = 6.23)	4.75 (Q1 = 3.88, Q3 = 5.91)	4.99 (Q1 = 4.26, Q3 = 5.96)
Males	6	4.24 (Q1 = 3.92, Q3 = 5.31)	4.72 (Q1 = 4.40, Q3 = 6.37)	5.42 (Q1 = 4.70, Q3 = 7.25)	4.99 (Q1 = 4.67, Q3 = 5.22)
Females	6	4.09 (Q1 = 3.66, Q3 = 5.14)	3.77 (Q1 = 3.56, Q3 = 5.61)	4.15 (Q1 = 3.71, Q3 = 4.77)	5.05 (Q1 = 4.03, Q3 = 6.43)
SARS-CoV-2 naïve	10	5.43 (Q1 = 4.12, Q3 = 5.88)	N/A	N/A	N/A
Males	0	N/A	N/A	N/A	N/A
Females	10	5.43 (Q1 = 4.12, Q3 = 5.88)	N/A	N/A	N/A

Data analyzed using a Friedman test for within the SARS-CoV-2 positive group analyses and using a multi-variate analysis of variance for between the SARS-CoV-2-positive and SARS-CoV-2-naïve group analyses. All data presented as median (Q1, Q3). *N* = number of participants, Q1 = 1st quartile, and Q3 = 3rd quartile.

NfL, neurofilament light; N/A, not available.

**Table 5.** GFAP Plasma Concentration at Each Visit

		GFAP (pg⋅mL^−1^)			
Group	*N*	Visit 1	Visit 2	Visit 3	Visit 4
SARS-CoV-2 positive	12	77.86 (21.49)	78.26 (23.71)	78.00 (25.39)	80.49 (21.23)
Males	6	75.36 (24.79)	70.81 (22.14)	77.48 (23.14)	74.47 (23.81)
Females	6	80.36 (19.66)	85.71 (24.77)	78.52 (29.71)	86.50 (18.37)
SARS-CoV-2 naïve	10	78.77 (21.74)	N/A	N/A	N/A
Males	0	N/A	N/A	N/A	N/A
Females	10	78.77 (21.740	N/A	N/A	N/A

Data analyzed using a repeated-measures analysis of variance for within the SARS-CoV-2-positive group analyses and using a multi-variate analysis of variance for between the SARS-CoV-2-positive and SARS-CoV-2-naïve group analyses. All data presented as mean (SD). *N* = number of participants.

GFAP, glial fibrillary acidic protein; SD, standard deviation; N/A, not available.

**Table 6.** Tau Plasma Concentration at Each Visit

		Tau (pg⋅mL^−1^)			
Group	*N*	Visit 1	Visit 2	Visit 3	Visit 4
SARS-CoV-2 positive	12	2.81 (1.21)	3.46 (1.38)	3.38 (1.32)	2.76 (1.14)
Males	6	2.81 (1.21)	3.03 (0.97)	2.60 (1.14)	2.67 (0.93)
Females	6	2.80 (1.46)	3.90 (1.67)	4.15 (1.05)	2.85 (1.41)
SARS-CoV-2 naïve	10	3.49 (1.51)	N/A	N/A	N/A
Males	0	N/A	N/A	N/A	N/A
Females	10	3.49 (1.51)	N/A	N/A	N/A

Data analyzed using a repeated-measures analysis of variance for within the SARS-CoV-2-positive group analyses and using a multi-variate analysis of variance for between the SARS-CoV-2-positive and SARS-CoV-2-naïve group analyses. All data presented as mean (SD). *N* = number of participants.

SD, standard deviation; N/A, not available.

**FIG. 3.**
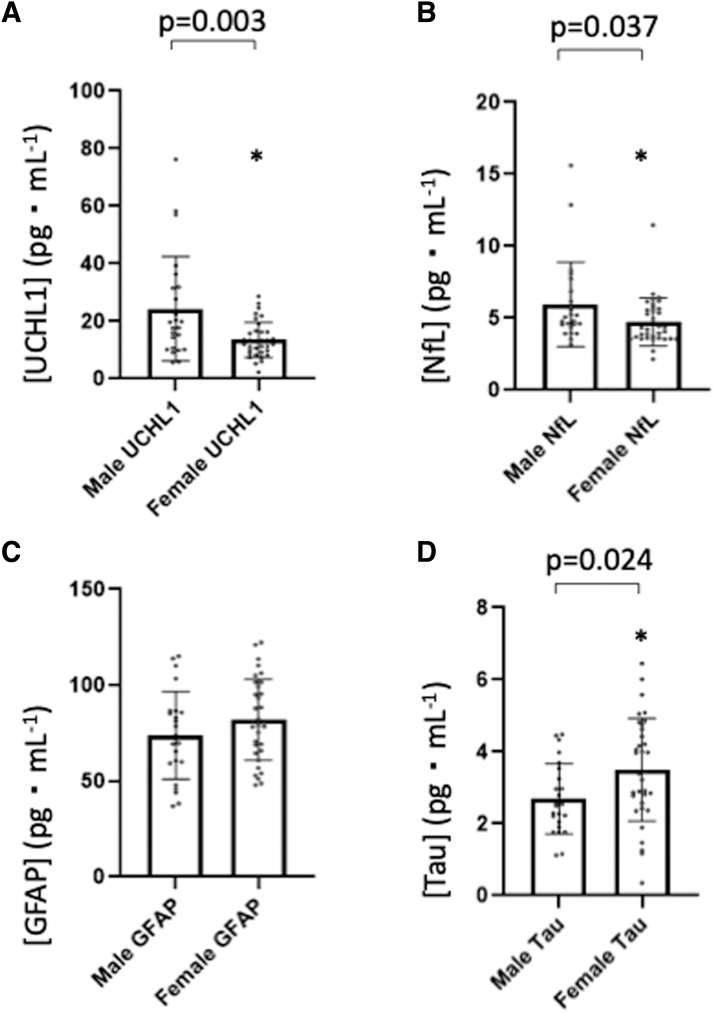
Bar graphs presenting male biomarker compared to female biomarker plasma concentrations. All female data presented are combined from both the SARS-CoV-2-positive and SARS-CoV-2-naïve groups. Data are presented as mean (SD). (**A**) Ubiquitin carboxyl-terminal esterase L1 (UCHL1) plasma concentration between males and females. (**B**) Neurofilament light (NfL) plasma concentration between males and females. (**C**) Glial fibrillary acidic protein (GFAP) plasma concentration between males and females. (**D**) Tau plasma concentration between males and females. *Significantly different (*p* < 0.005) compared to male participants.

MANOVA analysis was used to compare means of UCHL1, NfL, GFAP, and tau plasma concentration between visits from the SARS-CoV-2-positive group and the SARS-CoV-2-naïve groups, which showed no significant differences between visits (*F*_(16,196)_ = 0.406, *p* = 0.771, η^2^ = 0.056), nor was there a significant visit*sex interaction effect (*F*_(12,144)_ = 0.542, *p* = 0.884, η^2^ = 0.043). However, there was a significant difference between sexes regardless of diagnosis, that is, when female data from the SARS-CoV-2-positive and SARS-CoV-2-naïve groups were combined and compared to male data (*F*_(4,46)_ = 6.871, *p* < 0.001, η^2^ = 0.374). Tests of between-participants effects showed that significance occurred between sexes for UCHL1 (*F*_(4,46)_ = 8.396, *p* = 0.003, η^2^ = 0.146) with females (mean = 13.57 pg⋅mL^−1^, SD = 6.18) having a lower plasma UCHL1 concentration than males (mean = 24.34 pg⋅mL^−1^, SD = 18.09), NfL (*F*_(4,46)_ = 5.245, *p* = 0.037, η^2^ = 0.097) with females (mean = 4.75 pg⋅mL^−1^, SD = 1.66) having a lower plasma NfL concentration than males (mean = 5.97 pg⋅mL^−1^, SD = 2.92), and tau (*F*_(4,46)_ = 4.781, *p* = 0.024, η^2^ = 0.089) with females (mean = 3.50 pg⋅mL^−1^, SD = 1.43) having a higher plasma tau concentration than males (mean = 2.69 pg⋅mL^−1^, SD = 0.98). There were no significant differences in GFAP concentration (*p* = 0.176) between males and females. Combined female data from both the SARS-CoV-2-positive and SARS-CoV-2-naïve groups compared to male data are presented in Figure 3.

## Discussion

Our results showed no difference between SARS-CoV-2-positive participants and SARS-CoV-2-naïve participants for the plasma concentrations of UCHL1, GFAP, NfL, or tau. Within the SARS-CoV-2-positive participant group, there were no significant differences in plasma biomarker concentration among visits with the exception of UCHL1. For UCHL1, there was a higher plasma concentration of UCHL1 at visit 3 compared to visits 1 and 2. We also found sex differences for plasma UCHL1, GFAP, and tau only when female data from both the SARS-CoV-2-positive and SARS-CoV-2-naïve groups were combined and compared to the male data. Sex differences were not found when comparing male and female data within the SARS-CoV-2-positive group or between the male data from the SARS-CoV-2-positive group and female data from the SARS-CoV-2-naïve group.

We included analysis of plasma UCHL1 in our study because UCHL1 has been shown to indicate moderate TBI^39^ and has been found to be elevated in COVID-19 intensive care unit (ICU) patients,^31^ although UCHL1 has been less successful in distinguishing mild TBI.^40,41^ Despite this, UCHL1 was the only plasma biomarker that showed significant differences among visits within the SARS-CoV-2-positive participants, with plasma UCHL1 concentration at visit 3 being significantly greater than UCHL1 concentrations at visits 1 and 2. This is a unique finding given that our study is the first to investigate plasma UCHL1 in a cohort of participants infected with SARS-CoV-2 exhibiting only mild symptoms. This finding was surprising given that there was no significant difference between plasma UCHL1 concentrations at any visit and the SARS-CoV-2-naïve participants. However, it is possible that subtle changes in plasma UCHL1 caused by SARS-CoV-2 were more detectable in SARS-CoV-2-positive participants because the data are paired. It is important to note that visit 3 was the visit in which the most participants (4 of 12 or 33.33%) reported neurological symptoms (Table 1).

Our study found sex differences in plasma biomarker concentrations when female data from the SARS-CoV-2-positive and SARS-CoV-2-naïve groups were combined and compared to male data, with females having lower UCHL1 and NfL levels but higher tau levels than males. In agreement with our study, lower levels of serum UCHL1 in females compared with males have been found previously,^40,42^ along with no sex differences for GFAP.^42^ In contradiction to our findings, previous research has not found sex differences for serum tau.^42^ However, the assay in that study combined normal and phosphorylated tau as one measurement,^42^ whereas our study only measured normal tau, not phosphorylated tau, possibly causing the difference in findings. Interestingly, a study investigating cerebrospinal fluid (CSF) tau levels in Alzheimer's disease patients did find higher tau levels in females compared to males,^43^ which is in line with our findings. Also in agreement with our plasma NfL findings, studies investigating NfL levels in the CSF of patients with neurodegenerative diseases have found that women have lower CSF levels of NfL than men.^43,44^ However, in contradiction to our findings, serum and plasma NfL levels were found not to be different between sexes in frontotemporal dementia,^45^ Alzheimer's disease,^46^ inherited peripheral neuropathies patients,^47^ and elderly persons.^48^ This difference is surprising given that CSF NfL levels have been found to be highly correlated with plasma NfL levels,^49^ but may be attributable to disease and age differences between our participant population and these previous studies.

Overall, there has been a small amount of research conducted looking at brain injury biomarker differences in sex, given that sex is typically controlled for statistically as a confounding variable instead of investigating whether sex differences exist.^50^ Our study suggests that sex differences may need to be taken into consideration in future biomarker studies. However, it is important to note that we did not find sex differences when comparing male and female data within the SARS-CoV-2-positive group. The sex differences only appeared after combining data from the SARS-CoV-2-positive and SARS-CoV-2-naïve groups.

Previous research investigating TBI^51–53^ and COVID-19^25,27,31^ have found increases in all four biomarkers included in this study within hours to 30 days after injury or diagnosis. Therefore, if changes were to occur in these mildly symptomatic SARS-CoV-2-positive participants, we would have expected to observe these changes at some point over the course of the four visits. However, we did not find significant plasma UCHL1, GFAP, NfL, or tau differences in SARS-CoV-2-positive participants at any visit compared to SARS-CoV-2-naïve participants. Although our study is unique in examining these biomarkers in mildly symptomatic SARS-CoV-2-positive young adults, our results agree with three other studies which found that serum and plasma GFAP and NfL were not significantly different in healthy controls compared to COVID-19 patients with mild symptoms^23,26,29^ and also agrees with studies that have not found significant differences of plasma and serum tau^31,32^ in COVID-19 patients compared to healthy controls. Only one other study has investigated UCHL1 and found significantly higher arterial UCHL1 in COVID-19 participants upon hospital admission compared to healthy controls.^31^ Our study disagrees with this finding in that we did not find any differences in UCHL1 between SARS-CoV-2-positive and SARS-CoV-2-naïve participants. This may be because the study finding significant differences in UCHL1 included COVID-19 participants admitted to the ICU,^31^ whereas all our COVID-19 participants were not admitted to the hospital because they were mildly symptomatic. The difference in UCHL1 findings may also be because arterial blood was taken on the day of admittance to the ICU in that study,^31^ whereas venous blood was taken from our participants starting 3–4 weeks after COVID-19 diagnosis.

There are several limitations to this study. Not having paired SARS-CoV-2-naïve participants with the SARS-CoV-2-positive group was a limitation given that subtle differences would have been easier to detect, which is important in SARS-CoV-2-positive participants experiencing mild symptoms. However, it is almost impossible to have data from participants pre-pandemic and then obtain blood samples from them after contracting COVID-19 given that the pandemic was such a surprising event and human participant research was prohibited for some time after the pandemic began. We also had a small sample size with only 10 SARS-CoV-2-naïve controls and 12 SARS-CoV-2-positive participants, although we believe that this number of participants served the purpose of this study, which was simply to investigate the possibility of biomarker differences in these two groups before deciding whether conducting a larger-scale study was needed. Another limitation was the lack of males in the SARS-CoV-2-naïve participants. It would have been helpful to have had an equal number of males and females in the SARS-CoV-2-naïve group as we did in the SARS-CoV-2-positive group in order to allow better sex comparisons. However, data from the SARS-CoV-2-naïve group were collected just before the COVID-19 pandemic, stopping human subjects research and preventing us from obtaining male data. Despite these limitations, we did accomplish our goal of determining whether there were significant plasma biomarker differences between the SARS-CoV-2-naïve and SARS-CoV-2-positive participants worth further investigation.

## Conclusion

The purpose of our study was to determine whether the plasma biomarkers NfL, GFAP, UCHL1, or tau were higher in young adult participants with mild COVID-19 symptoms compared to young adult SARS-CoV-2-naïve participants, given that there is evidence of these biomarkers being elevated in older adults with moderate-to-severe COVID-19 symptoms. Our data showed no differences between plasma biomarker concentrations in the SARS-CoV-2-positive participants compared to the SARS-CoV-2-naïve participants. However, within the SARS-CoV-2-positive participants, UCHL1 was significantly higher at visit 3 compared to visits 1 and 2; and visit 3 also corresponded to the greatest number of neurological symptoms reported. There were sex differences in three of the four biomarkers only when female data from the SARS-CoV-2-positive and SARS-CoV-2-naïve groups were combined and compared to male data, with women having a lower plasma UCHL1 and NfL concentration and a higher plasma tau concentration. This indicates that sex differences may need to be taken into consideration in future COVID-19 biomarker studies. Although sex differences were found in our small population, it does not appear that young healthy persons with mild COVID-19 symptoms experience changes in the brain injury biomarkers NfL, GFAP, tau, or UCHL1.

## Supplementary Material

Supplemental data

## Abbreviations Used

COVID-19: corona virus disease 2019
CSF: cerebrospinal fluid
GFAP: glial fibrillary acidic protein
ICU: intensive care unit
MANOVA: multi-variate analysis of variance
NfL: neurofilament light
RMANOVA: repeated-measures analysis of variance
SARS-CoV-2: severe acute respiratory syndrome coronavirus 2
SD: standard deviation
TBI: traumatic brain injury
UCHL1: ubiquitin carboxyl-terminal esterase L1
