# Supplementary material for: Plasma UCHL1, GFAP, Tau, and NfL Are Not Different in Young Healthy Persons With Mild COVID-19 Symptoms Early in the Pandemic: A Pilot Study
Source: Neurotrauma Rep. 2023 May 15;4(1):330–41. doi: 10.1089/neur.2023.0014 (PMC10240333; doi:10.1089/neur.2023.0014)
Supplement: Supplemental data [file Suppl_TableS1.docx]

| Variable | Reporting  n SARS-CoV-2 Naïve; n SARS-CoV-2 Positive | SARS-CoV-2 Naïve | SARS-CoV-2 Positive |
| --- | --- | --- | --- |
| Race/Ethnicity  White n,(%)  Black n,(%)  Asian n,(%) | 10; 12 | 8, (80%)  1, (10%)  1, (10%) | 12, (100%)  0, (0%)  0, (0%) |
| Health History  Asthma  Bronchitis  Anemia | 10; 12 | 1, (10%)  1, (10%)  2, (20%) | 2, (16.7%)  2, (16.7%)  1, (8.3%) |
| Allergies  Seasonal  Animal  Food  Medication | 10; 12 | 2, (20%)  2, (20%)  3, (30%)  4, (40%) | 1, (8.3%)  0, (0%)  2, (16.7%)  2, (16.7%) |
| Medications  Birth Control  Asthma  Anemia  Depression/Anxiety | 10; 12 | 6, (60%)  1, (10%)  1, (10%)  2, (20%) | 5, (41.7%)  0, (0%)  1, (8.3%)  3, (25%) |
| Family History  Diabetes  High Blood Pressure  Heart Disease  Obesity  Cancer  Asthma  Arthritis  Osteoporosis | 10; 12 | 2, (10%)  4, (40%)  0, (0%)  1, (10%)  2, (20%)  2, (20%)  1, (10%)  1, (10%) | 2, (16.7%)  4, (33.3%)  3, (25%)  2, (16.7%)  4, (33.3%)  3, (25%)  0, (0%)  0, (0%) |

Table S1: Race/Ethnicity data along with health history of the participants in this study. Data are presented as the number and percentage of participants each category applies to.
